# Cultural transmission and religious belief: An extended replication of Gervais and Najle (2015) using data from the International Social Survey Programme

**DOI:** 10.1371/journal.pone.0305635

**Published:** 2024-06-24

**Authors:** Tatsunori Ishii, Katsumi Watanabe

**Affiliations:** 1 Department of Psychology, Faculty of Integrated Arts & Social Science, Japan Women’s University, Tokyo, Japan; 2 Faculty of Science and Engineering, Waseda University, Tokyo, Japan; 3 University of New South Wales, Sydney, Australia; Max Planck Institute for Evolutionary Anthropology, GERMANY

## Abstract

Beliefs in supernatural agents or religious beliefs are pervasive, yet there are individual differences in such beliefs. Although various factors have been proposed as relevant, recent research has increasingly emphasized the importance of cultural learning, showing that enthusiastic religious behavior (credibility enhancing displays; CREDs) from parents predicts increased religious beliefs among their children. In addition to this kin-biased learning, Gervais and Najle (2015) analyzed data from the World Values Survey to demonstrate that the number of adults who show religious CREDs is also an important predictor of people’s beliefs, indicating that individuals develop their religious beliefs through conformist learning. This pre-registration study aimed to replicate and extend these findings by analyzing data from the International Social Survey Programme (ISSP), which is another large social survey. We examined the generalizability of the results by analyzing multigenerational samples. Multilevel regression and signal detection analyses revealed that the presence of both kin-biased and conformist learning cues was significantly associated with respondents’ religious beliefs. Moreover, they suggested tension between the two cultural learning cues, thereby suggesting that the effect of kin-biased learning on religious beliefs becomes stronger (weaker) when the cue for conformist learning is unclear (clear). These results support the idea that these two types of cultural learning are crucial to the development of religious beliefs.

## 1. Introduction

Currently, the belief in supernatural beings, such as gods and spirits, is attested worldwide and has been this way throughout history. However, there are individual differences in such religious beliefs, with some people strongly committed to it and others less so. Why are religious beliefs so pervasive, yet there are clear individual differences? Various factors that could answer this question have been proposed by many scholars, such as psychologists, anthropologists, sociologists, and cognitive scientists of religion. This article focused on one of such factors, credibility enhancing displays (CREDs) in cultural transmission, and it examined the relationship between religious CREDs and religious beliefs by analyzing data from a large social survey, the International Social Survey Programme (ISSP). We first summarize the potential factors associated with the diversity in religious beliefs, after which we discuss the details of this relationship.

### 1.1. Potential factors associated with religious beliefs

#### Cognitive abilities

Scholars in the cognitive science of religion argue that the cognitive abilities of human beings evolved in the ways that underpin religious belief. For example, Barrett and his colleagues argued that humans are equipped with a sensitive agency-detection system for evolutionary reasons (i.e., to detect predators reliably, even at the risk of a false positive), and this “hyperactive or hypersensitive agency-detection device” enables people to perceive the existence of invisible agents (e.g., [1, 2]). Likewise, the presence of such perceived agents would be significantly stronger owing to human beings’ mentalizing ability or theory of mind. People who attribute mental states to such agents recognize them as having the ability to think, feel, and communicate with humans (e.g., [3]).

According to this argument, these cognitive abilities are associated with the individual differences in religious beliefs. Indeed, previous studies have shown that, for instance, people with a high mentalizing ability have stronger religious beliefs (e.g., [4–6]). Moreover, it has also been demonstrated that thinking styles that foster or inhibit the use of these cognitive abilities are related to the degree of believing in gods. Specifically, many works showed that people who employ analytic thinking as a dominant style were less religious than intuitive thinkers (e.g., [7–9]). However, the strength of the association between cognitive abilities (e.g., mentalizing) and religious beliefs is not as strong as previously assumed (e.g., [8, 10–12]). This may be because this cognitive approach explains how people can represent supernatural agents, but less so why people consider these agents to be true [13, 14].

#### 1.1.2. Cultural transmission and CREDs

It has long been recognized that people change their beliefs, opinions, and behaviors as a result of teaching, imitation, and other forms of social learning (e.g., [15]). Similarly, religious beliefs are transmitted culturally. Researchers on cultural evolution [14, 16, 17] have identified three mechanisms of such transmission: content-based, context-based, and CREDs. The content-based mechanism refers to the idea that cultural information of a specific content type (e.g., emotionally evocative ideas, minimally counterintuitive concepts) is likely to be transmitted. Minimally counterintuitive concepts are those with content that minimally violates people’s intuitive ontological knowledge (e.g., folk biology, physiology, and psychology). This concept is considered one of the cognitive underpinnings of religious concepts, but it has also been argued that it has the advantage of being easier to remember, and therefore easier to transmit (e.g., [18, 19]).

The context-based mechanism describes the person bringing the information to be transmitted. People are likely to learn cultural information from models that are similar to them, are successful, and have a social status. Learning from information that is observed frequently (conformist learning) and learning from family (kin-biased learning) are also part of the context-dependent mechanism.

CREDs are “displays (often actions) that indicate a model’s degree of commitment to, or belief in, verbally expressed representations” [14 p. 258]. According to the theory of CREDs, humans tend to learn cultural information, such as beliefs and opinions, from others via verbal communication, and there is a risk of being persuaded to learn false information. Thus, humans must have evolved defense mechanisms to avoid being verbally manipulated by self-interested cultural models who express beliefs or opinions to which they do not commit (see also [20]). Attending to CREDs is such a mechanism; people could diagnose verbally expressed beliefs as true if models showed behaviors that would appear costly if they had a belief that is at odds with those behaviors (CREDs). In other words, “without CREDs, learners are not committed to those recalled representations in a manner that propels behavior beyond simply repeating the expression itself” [14 p. 247]. It has been argued that CREDs work in the transmission of various cultural information, such as food preferences, opinions, and altruism (e.g., [21]). In the case of religion, religious beliefs often involve costly behaviors, such as frequent attending to religious services or extreme rituals. For such a costly behavior-belief set to be transmitted and to persist, CREDs are essential.

Early empirical works in the cognitive science of religion focused on content-based mechanisms (e.g., [18]), but recent studies have increasingly shown the importance of context-based mechanisms and CREDs in explaining to what extent people commit to religious beliefs. For example, Lanman and Buhrmester (2017) [22] developed a scale that assessed the extent to which the respondents were exposed to their caregivers’ religious CREDs (e.g., “To what extent did your caregiver(s) attend religious services or meetings?”) and demonstrated a score for the CREDs exposure scale to explain the individual differences in the belief in gods, even after controlling for religious socialization (i.e., to what extent the respondents’ important people talked about or taught religion with emphasis during their upbringing). This suggests that even if parents are religious, children are not necessarily religious unless parents show religious CREDs. More importantly, it suggested that people employ their parents’ CREDs over verbal communication to what to learn, even if parents are generally less likely to manipulate their children. Other previous studies have also shown the same line of evidence [8, 12, 23, 24]. The positive association between parents’ religious beliefs and those of their children [25–27] may support the importance of caregivers’ religious CREDs, albeit indirectly.

#### 1.1.3. Other factors

Other factors that account for the individual differences in religious belief have also been investigated (e.g., [28]). For instance, secularization theory suggests that religion works as a “tranquilizer” for existential concerns, such as fear of death, and thus, religious belief declines as existential security increases. Supporting this prediction, it has been shown that people who live in countries with economic development, income security, and health security are less religious (e.g., [29, 30]). Gervais et al. (2021) [8] also showed that, in the United States, people feeling existentially secure tend to be atheists. Other works have pointed out that religiosity works as a mating strategy; religiosity is often associated with sexually conservative behavior, and men and women who pursue monogamous, high-fertility strategies in mating tend to be religious (e.g., [31]).

### 1.2. Focusing on CREDs

Among the factors mentioned above, this study focused on cultural transmission and CREDs. There were some reasons for this. As described above, the original CREDs paper [14] suggested that CREDs are important in the transmission of various cultural information, but it was not so long ago that the relationship between CREDs and religious belief was empirically examined (e.g., [22]). It is important to accumulate more empirical evidence about this relationship. In addition, nowadays, CREDs are considered promising factors that explain the individual differences in religious beliefs. For example, only the CREDs theory offers a formal model for why people are deeply committed to religious beliefs and devote themselves to costly practices. Indeed, previous studies revealed that exposure to parents’ religious CREDs is a better predictor of religious belief than cognitive abilities [8, 12] or other socio-emotional dispositions (i.e., empathic concern) [24] and secularization [8]. It is worth examining this relationship between CREDs and belief for a deeper understanding of religious diversity worldwide (e.g., increasing Atheists).

#### 1.2.1. Kin-biased and conformist learning

In the literature on CREDs and religious beliefs, previous studies (e.g., [8, 12, 22, 24]) mainly examine kin-biased learning (i.e., caregivers’ religious CREDs). This is reasonable because it is no surprise that people imitate and learn from others who have been with them for a long time since infancy. Simultaneously, people tend to use CREDs over verbal approaches [14]. Therefore, parents’ CREDs should have a long-term impact on their children’s beliefs.

On the other hand, other cultural learning pathways have not been as well examined in this literature. For example, although many findings in social psychology studies suggest that social behavior is significantly influenced by the behavior of the majority in a society (i.e., conformity), conformist learning has rarely been examined in the case of religious beliefs. This is partly because of difficulties in detecting frequency-dependent effects on religious beliefs (e.g., estimating the number of people engaging in religious behavior) with high precision using a standard survey method.

Gervais and Najle (2015) [32] demonstrated the conformist learning of religious beliefs via CREDs by analyzing data from the World Values Survey (WVS), which is an international research program investigating the different values held by people worldwide since 1981 [**Note 1**]. From the WVS dataset, they sampled two groups with different birth years (one group involved people born between 1971 and 1981, and the other group involved people born before 1970) to determine whether the number of people in the older group who showed eager religious behavior predicted religious beliefs among those in the younger group. They established that younger respondents who lived in a country or region with a higher percentage of religiously eager older respondents were highly likely to believe in gods, thereby suggesting that the religious CREDs of older adults evoked conformist responses from younger people through conformist learning. This association was statistically independent of the extent to which individual differences in religious beliefs in the younger group could be explained through their retrospective answers regarding whether they were raised as religious people at home (kin-biased learning).

Apart from this individual-level analysis, it should be noted that Gervais and Najle (2015) [32] also achieved similar results in country- or international-level analyses. Based on the signal detection theory, Gervais and Najle (2015) [32] regarded the kin-biased learning cue (religious upbringing) as a signal, and they regarded the country or region as a unit. Through this approach, they calculated both the response sensitivity (*d’*, which indicates the degree of accuracy with which a signal is detected and responded to) and response bias (*C*, which indicates the frequency of people’s tendencies to respond to a signal, regardless of whether it is present) levels of the focal group. If people are sensitive to religious upbringing and thus believe in gods in general, the average sensitivity index *d’* across countries differs from the chance levels significantly. Moreover, if people tend to conform to the behaviors of other individuals around them, they eventually believe in gods, regardless of any religious upbringing. Consequently, the bias index *C* is correlated with the percentage of religiously eager people in the older group. Gervais and Najle (2015) [32] showed that the results were precisely in line with both predictions. These findings, which overcame the limitations of a standard survey, suggested that religious beliefs are transmitted through conformist learning and kin-biased learning.

### 1.3. The current study

This pre-registration study (https://osf.io/kdbm7) aims to replicate and extend Gervais and Najle (2015)’s [32] findings in terms of CREDs. We examined whether religious CREDs of caregivers (i.e., kin-biased learning) and older adults (i.e., conformist learning) explain the individual differences in religious beliefs. In this study, we analyzed data from the International Social Survey Programme (ISSP), which is another large social survey. Similar to the WVS, the ISSP is a cross-cultural study. It was established in 1984, and it covers various topics, including religion. Its large dataset has been used in many social science studies. The ISSP was chosen because its questionnaire [33] includes items that can be used to create religious CREDs for caregivers, which the WVS did not have. It should also be noted that the ISSP differs from the WVS in terms of the countries and regions surveyed, as well as the sampling methods, question items, and the wording of items, among other aspects. Consequently, investigating the same hypothesis using data from the WVS and those from the ISSP sometimes yields different results (e.g., [34]). Therefore, the ISSP dataset is helpful in testing the external validity or generalizability of Gervais and Najle (2015)’s [32] findings.

According to Gervais and Najle (2015) [32], we created a cue for conformist learning; the religious CREDs of older adults. Using an item asking how often the respondents participated in religious activities or organizations, we calculated the percentage of respondents in the older group who participated weekly or more than once per week in each region or country. If people are highly likely to learn religious beliefs depending on the number of people who engage in enthusiastic religious behavior (i.e., religious CREDs), this cue would be associated with the religious beliefs of respondents in the younger group.

We also created cues for kin-biased learning (the religious CREDs of caregivers) using similar items as the conformist learning cues; items asking about the frequency of parents’ attendance at religious services during childhood. These items were used because the current study focused on CREDs in the transmission of religious beliefs, although the original study [32] used an item on religious upbringing as the kin-biased learning cue. Note that this item is similar to one of the items on the CREDs exposure scale (“To what extent did your caregiver(s) attend religious services or meetings?” [22]), and it is a valid indicator of religious CREDs. As mentioned above, previous studies have already shown that exposure to caregivers’ religious CREDs is associated with individual differences in religious beliefs. Thus, a similar result was predicted. However, those attempts were conducted mainly in Western countries and rarely in other cultural areas. Therefore, this study will provide preliminary evidence on global trends in the relationship between caregivers’ religious CREDs and religious beliefs.

Furthermore, taking advantage of the fact that the ISSP contains questions about both fathers’ and mothers’ religious behavior, we conducted an exploratory examination of which parents’ religious CREDs were more influential, despite concerns that parents’ behaviors would be correlated. Cavalli-Sforza et al. (1982) [25] reported that mothers’ religiosity (e.g., whether they are affiliated with Christianity or Judaism and whether they attend a church) was more strongly correlated with their children’s religiosity than fathers’ religiosity. This finding might suggest that mothers are highly influential in the religious domain. However, their survey was conducted almost four decades ago and only in the United States, and more importantly, it was not conducted in terms of CREDs. Hence, we could not make clear predictions.

The same analytical strategies implemented by Gervais and Najle (2015) [32] were used to replicate their findings. We first employed multilevel modeling using the two types of cultural learning cues to predict individual differences in religious beliefs by considering a data structure in which each respondent was nested in a country or region. We utilized an international-level analytical approach based on the signal detection theory to establish whether people had increased response sensitivity to the signals around them (i.e., whether their religious beliefs were in response to their parents’ CREDs). We also conducted analyses to determine whether their response bias levels were associated with the frequency of the religious behaviors shown by the people around them (i.e., people living in a country or region where the value of the cue for conformist learning is high tend to have strong religious beliefs).

We also examined the robustness of the findings by analyzing data from other generations of respondents. As noted above, Gervais and Najle (2015) [32] isolated respondents born between 1971 and 1981 as the focal group and those born before 1970 as the older group. However, other focal and older groups could be created (e.g., respondents born between 1981 and 1991 could represent another focal group), and we investigated whether similar results would be obtained after the birth year condition was varied.

## 2. Methods

### 2.1. Samples

ISSP: Religion III [33] included data from 59,982 respondents from 43 countries or regions, ranging in age from 15 to over 98 years. After excluding respondents who provided missing values (e.g., no answer, “can’t choose,” “can’t say”) for the target questions below, we isolated a subsample of respondents who were born between 1971 and 1981 (the main focal group, *N* = 8900) and another subsample of respondents born before 1970 (the main older group, *N* = 31,788) **[Note 2]**.

### 2.2. Measures

#### 2.2.1. Religious beliefs

Following the work of Gervais and Najle (2015) [32], we selected an item that directly asked whether the respondents believed in God or gods (Q16; “Please indicate which statement below comes closest to expressing what you believe about God”) as the primary measure of religious belief in the focal group. Six non-continuous (qualitatively different) responses were possible for this item (e.g., 1 = “I don’t believe in God,” 2 = “I don’t know whether there is a God and I don’t believe there is any way to find out,” 3 = “I don’t believe in a personal God, but I do believe in a Higher Power of some kind,” 4 = “I find myself believing in God some of the time, but not at others,” 5 = “While I have doubt, I feel that I do believe in God,” 6 = “I know God exists and I have no doubts about it”). Next, we dichotomized these responses to match the methodology employed by Gervais and Najle (2015) [32] and to enable signal detection analysis. We assigned a score of 0 (absent) for choices from 1 to 3 because these choices included the words “I don’t believe in God.” Similarly, we gave a score of 1 (present) to the respondents whose choices were from 4 to 6, which included the words “I believe in God.”

We also assessed religious beliefs in the focal group using an item asking participants about the extent to which they described their religiousness (Q31). As the choices for this item ranged from 1 (“Extremely religious”) to 7 (“Extremely non-religious”), we regarded this item as a 7-point Likert scale and used the rating (1 to 7) as another score for religious belief. In the analyses, we reversed the score, such that a higher score indicated stronger religious beliefs.

#### 2.2.2. Kin-biased learning cues

The following items were included in the ISSP: “When you were a child, how often did your mother attend religious services?” (Q24) and “When you were a child, how often did your father attend religious services?” (Q25). The answers to these items (1 = never; 9 = several times a week) in the focal group were used to investigate kin-biased learning. Due to our focus on CREDs, we defined the presence of parents’ religious CREDs based on whether the respondents’ answers to the items were 8 or 9 (weekly or more than weekly), or any other response. We coded answers 1–7 to Q24 as 0 (mothers’ CREDs were absent) and answers 8–9 were coded as 1 (present). Similarly, we coded the answers to Q25 (0 = fathers’ CREDs were absent, 1 = present). Notably, items on the frequency of the mothers’ and fathers’ attendance were separated. This allowed us to determine the parents’ CREDs that were significantly associated with their children’s religious beliefs, which Gervais and Najle (2015) [32] did not investigate.

#### 2.2.3. Conformist learning cue

To investigate conformist learning, we used the responses (1 = never– 9 = several times a week) to the following item: “How often do you take part in the activities or organizations of a church or place of worship other than attending services?” (Q28). Following the work of Gervais and Najle (2015) [32], in each country or region, we calculated the percentage of respondents in the older group who participated weekly (8) or several times a week (9).

### 2.3. Analytical strategies

Following the work of Gervais and Najle (2015) [32], we employed two analytical approaches to determine whether kin-biased and conformist learning cues were associated with religious beliefs in the focal group.

#### 2.3.1. Individual-level analyses

As the respondents in our dataset were not statistically independent owing to the hierarchical structure of the data (i.e., each respondent was nested in a country or region), we assumed that the associations between kin-biased learning cues and the measure of religious beliefs varied depending on the country or region. Additionally, the conformist learning cue was a country- or region-level variable. Therefore, we employed multilevel modeling to test our predictions.

Following the work of Gervais and Najle (2015) [32], we conducted a stepwise hierarchical analysis. In the first step, we set the country or region as a random factor (i.e., level-2 residual) to examine how religious belief levels varied in terms of clusters. In the second step, we added the kin-biased learning cue as a fixed factor and as a random factor. The latter considered the kin-biased transmission of religious beliefs to differ between clusters (i.e., as a residual term associated with the level-1 predictor). In the third step, we added the conformist learning cue and the interaction between the two types of cues as fixed factors. These two cultural learning cues would explain religious beliefs if the beliefs were acquired through kin-biased and conformist learning. Gervais and Najle (2015) [32] found that the interaction was not statistically significant, and therefore, we anticipated the same result. We tested these predictions and explored which parents’ religious CREDs show a more robust association. Note that there was concern that the results of the analyses might be biased due to the likelihood of correlation between the two kin-biased tilt cues (i.e., multicollinearity). Therefore, we confirmed that this problem did not occur by calculating the VIF.

#### 2.3.2. International-level analyses

We also utilized an approach based on signal detection to investigate the association between the two types of cultural learning cues and religious beliefs. As described in **Table 1**, we treated parents’ religious CREDs as a signal (0 = absent, 1 = present) and the belief in gods measure as a response (0 = absent, 1 = present). Next, for each country or region, the sensitivity (*d’*) and bias (*C*) indices were calculated using the data from the focal group, following the standard procedure of signal detection theory [**Note 3**]. If kin-biased transmission occurred, people would be sensitive to their parents’ religious CREDs and come to believe in gods in general. Therefore, the average sensitivity index *d’* across countries would be different from the chance level (i.e., zero). Moreover, if conformist learning occurs, people who live in countries or regions with higher percentages of older adults who show religious CREDs would believe in gods, regardless of their parents’ religious CREDs. Therefore, the conformist learning cue would explain the bias index *C*. As in the individual-level analysis, we tested these predictions separately, depending on whether the cue came from the mother or father.

**Table 1:** The four outcomes of the approach based on signal detection theory.

	Signal: Parents’ Religious CREDs	
Response: Belief in gods	1 = Yes (present)	0 = No (absent)
1 = Yes (present)	Hit	False Alarm
0 = No (absent)	Miss	Correct Rejection

#### 2.3.3. Additional analyses

We also analyzed data from other generations of respondents to confirm the robustness of our findings. As described in section 2.1, our main focal group was the respondents born between 1971 and 1981. We also created other focal groups: younger and older. We subsampled the respondents born between 1981 and 1990 as the younger focal group, and those born before 1980 as the corresponding older group. Furthermore, the respondents born between 1961 and 1971 were subsampled as the older focal group, and those born before 1960 represented the corresponding older group. We investigated whether similar results would be obtained using younger and older focal groups, as well as their corresponding older groups.

## 3. Results

### 3.1. Individual-level analyses

First, following the analytical strategy, we conducted hierarchical multilevel modeling to test our predictions. The simple bivariable correlation matrix of the key variables is shown in **S1 Table**.

#### 3.1.1. Belief in gods

We conducted hierarchical multilevel logistic regression analysis using the two kin-biased learning cues (level-1 variables; 0 = parents’ CREDs were absent, 1 = present) and the conformist learning cue (level-2 variable; percentage of the respondents in the older group who showed religious CREDs in each country or region) to explain the belief in gods (0 = absent; 1 = present). We also entered gender (0 = male; 1 = female) into the models at all steps to statistically control for the association between gender and the belief in gods (i.e., females are more religious than males in general), although this association was not our focus. The conformist learning cue was standardized because the range of this variable was so narrow that its coefficients in the logistic regression model would be too large (>10) and impractical [**Note 4**].

In the first-step model, we identified a non-negligible amount of variation involving the belief in gods between different countries or regions. The random intercept variance was 1.369, which was significantly different from zero, according to a likelihood ratio test involving the corresponding model without the level-2 random factor (*χ*^2^ = 1820.62, *df* = 1, *p* < .001). The interclass correlation coefficient also indicated that approximately 30% of the chances of believing in gods were explained by inter-country differences (ICC = .294).

In the second step, we investigated the associations between the two kin-biased learning cues and the belief in gods together with the between-country variances of these associations **[Note 5]**. Therefore, in addition to the first-step model, the second model involved two kin-biased learning cues included as fixed factors, as well as two random slope terms associated with these cues. First, we investigated whether these random slope terms improved the corresponding model, which did not assume the random factors of the kin-biased learning cues, by entering them separately into the model. The deviance of the model with no random slope terms was 9002.8, and the deviance of the model with the random slope term associated with the mothers’ CREDs was 8994.7. This difference was statistically significant (*χ*^2^ = 8.07, *df* = 1, *p* = .004). Additionally, the model without random slope terms improved significantly when the term associated with the fathers’ CREDs was included (deviance = 8989.5, *χ*^2^ = 13.24, *df* = 1, *p* < .001). Therefore, we could justify the assumption of between-country variances in the associations between the two kin-biased learning cues and the belief in gods, after which we proceeded with the second step of the analysis. As we predicted, the second model showed that the presence of both the mothers’ CREDs (*b* = 0.61, OR = 1.85, 95% CI [1.46, 2.34], *z* = 5.14, *p* < .001, VIF = 1.19) and the fathers’ CREDs (*b* = 1.05, OR = 2.84, 95% CI [2.10, 3.85], *z* = 6.74, *p* < .001, VIF = 1.19) were associated with the individual differences in the belief in gods. The 95% CI indicated that respondents whose mother (father) showed religious CREDs had 1.46 to 2.34 (2.10 to 3.85) times higher chances of believing in gods. The fitness of this model (deviance = 8988.1) was significantly better than that of the model with no random slope terms (*χ*^2^ = 14.65, *df* = 2, *p* < .001), thereby indicating that the associations between the two kin-biased learning cues and the belief in gods varied by country or region.

In the third step, we included the conformist learning cue as an independent predictor and as an interaction term together with the two kin-biased learning cues (**Table 2**). This approach improved the model in the second step (deviance = 8963.7, *χ*^2^ = 24.40, *df* = 3, *p* < .001). The third step’s model demonstrated that the mothers’ CREDs (*b* = 0.59, OR = 1.81, 95% CI [1.43, 2.29], *z* = 4.93, *p* < .001, VIF = 1.22) and the fathers’ CREDs (*b* = 1.06, OR = 2.89, 95% CI [2.17, 3.86], *z* = 7.25, *p* < .001, VIF = 1.24) were associated with the belief in gods same as the second model. The conformist learning cue was also associated with the belief in gods (*b* = 0.69, OR = 1.99, 95% CI [1.50, 2.63], *z* = 4.80, *p* < .001). The 95% CI indicated that the average level of the belief in gods in each country was 1.50 to 2.63 times higher when the number of older adults who showed religious CREDs increased by 1 *SD*.

**Table 2:** The third-step model of hierarchical multilevel logistic regression analysis for the belief in gods in the main focal group.

		95% confidence interval			
Predictors	Odds ratio	Lower bound	Upper bound	*z*	*p*
Intercept	1.33	1.00	1.76	1.95	.051
Gender	1.65	1.49	1.84	9.49	< .001
Mother’s CREDs	1.81	1.43	2.29	4.94	< .001
Father’s CREDs	2.89	2.17	3.86	7.25	< .001
Conformist learning cue	1.99	1.50	2.63	4.80	< .001
Mother’s CREDs * Conformist	1.09	0.86	1.39	0.71	.479
Father’s CREDs * Conformist	0.69	0.52	0.92	–2.50	.012
Random intercept variance	0.79				
Random slope variance					
Mother’s CREDs	0.10				
Father’s CREDs	0.19				

**Fig 1:**
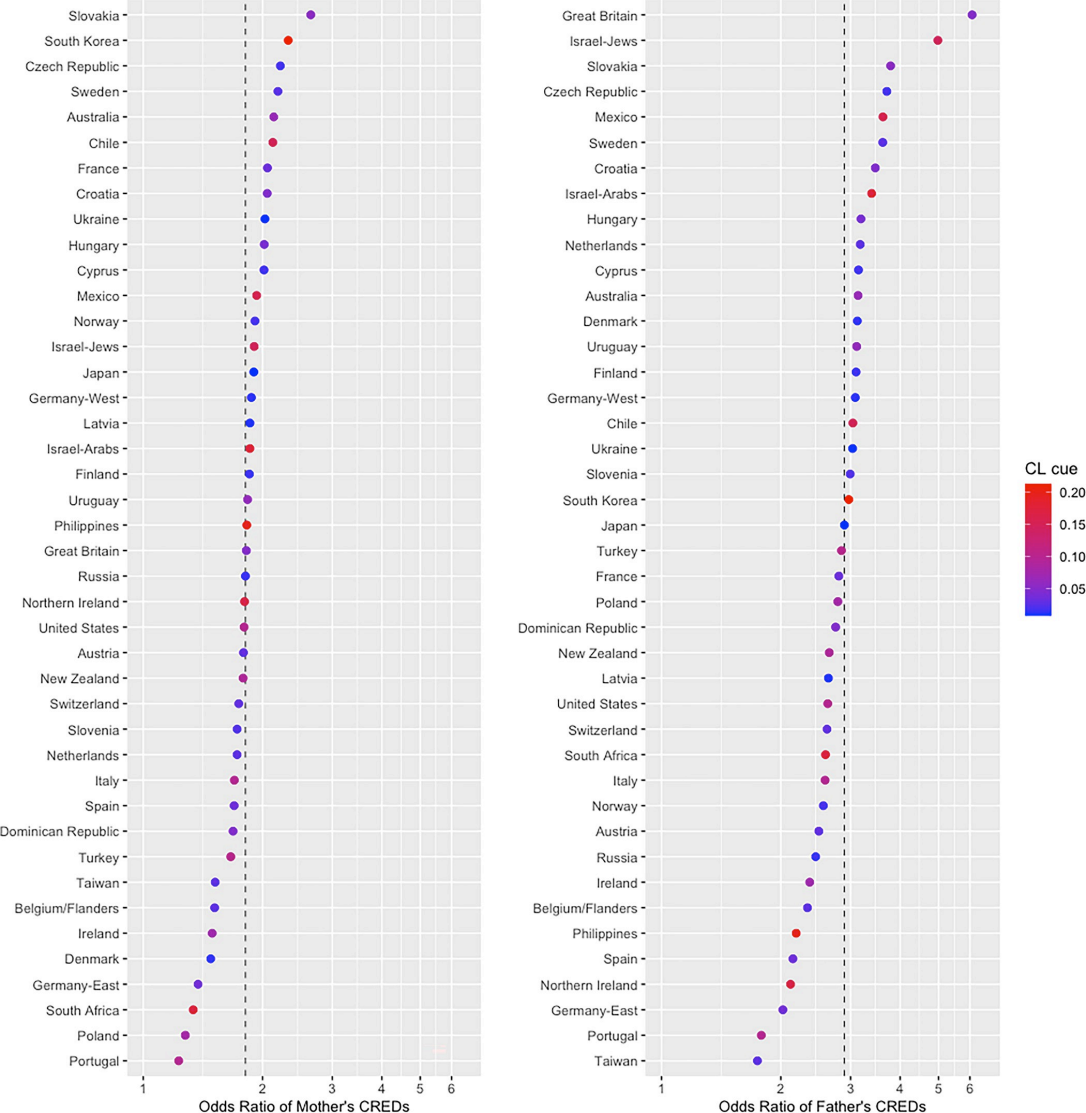
The association between kin-biased learning cues and the belief in gods (odds ratios) across 42 countries or regions in the analysis of the data from the main focal group. The dotted line indicates the fixed slope of the kin-biased learning cue in the overall sample. Color gradients indicate the conformist learning (CL) cue in each country or region. The horizontal axis indicates the odds ratio of kin-biased learning cues on a log scale.

As we anticipated, the interaction between the conformist learning cue and the mothers’ CREDs was unlikely (*b* = 0.09, OR = 1.09, 95% CI [0.86, 1.39], *z* = 0.71, *p* = .480) because the 95% CI was across zero (i.e., it is unclear that the association between mother’s CREDs and the belief in gods could be weakened or strengthened by a 1-point (1 *SD*) change in the conformist learning cue). However, the interaction between the conformist learning cue and the fathers’ CREDs were likely (*b* = −0.37, OR = 0.69, 95% CI [0.52, 0.92], *z* = −2.50, *p* = .012); the fixed slope of the father’s CREDs (*b* = 1.06) changed by –0.37 as the cultural learning cue increased by 1 *SD*. A simple slope analysis demonstrated that the association between the fathers’ CREDs and the belief in gods was weaker when the conformist learning cue was 1 *SD* higher (*b* = 0.69, OR = 2.00, 95% CI [1.38, 2.91], *z* = 3.64, *p* < .001) than when it was 1 *SD* lower (*b* = 1.43, OR = 4.18, 95% CI [2.70, 6.48], *z* = 6.41, *p* < .001). This suggested that the fathers’ religious CREDs resulted in increased chances of their children believing in gods among the countries or regions where fewer older adults exhibit religious CREDs. The odds ratios of the parents’ CREDs in each country or region are presented in **Fig 1**. The conformist learning cue for each country or region is indicated using different color gradients.

#### 3.1.2. Religiosity

We also conducted hierarchical multilevel linear regression analysis using the two kin-biased learning cues and the cue for conformist learning to determine the extent to which the respondents described their religiosity levels. This measure of religiosity was moderately associated with the measure of the belief in gods (correlation ratio: *η*^2^ = .577).

**Fig 2:**
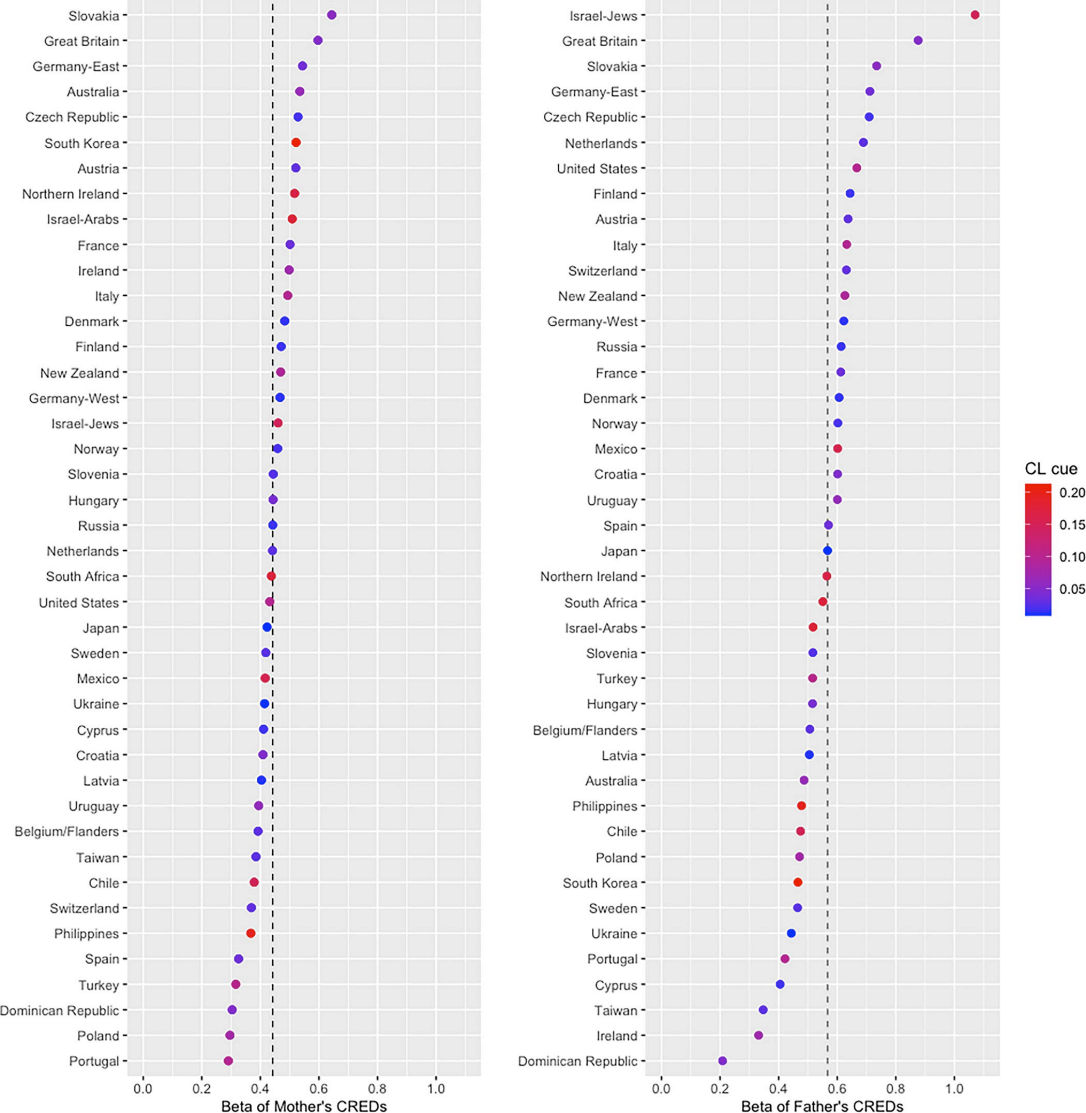
The association between kin-biased learning cues and religiosity (beta) across 42 countries or regions in the analysis of the data from the main focal group. The dotted line indicates the fixed slope of the kin-biased learning cue in the overall sample. Color gradients indicate the conformist learning (CL) cue in each country or region.

This analytical strategy was identical to that of the analysis involving the use of the measure for the belief in gods as an outcome variable. The first-step model indicated a significant amount of variation in religiosity levels between different countries or regions. The random intercept variance (0.476) was significantly different from zero (*χ*^2^ = 1787.2, *df* = 1, *p* < .001), and the ICC was .205. In the second step, both the mothers’ CREDs (*b* = 0.44, 95% CI [0.31, 0.56], *t* = 6.84, *p* < .001, VIF = 1.18) and the fathers’ CREDs (*b* = 0.55, 95% CI [0.40, 0.69], *t* = 7.56, *p* < .001, VIF = 1.18) were associated with religiosity. According to the 95% CI, the presence of mother’s (father’s) religious CREDs resulted in 0.31 to 0.56 (0.40 to 0.69) points higher in the religiosity score. The fitness of this second-step model was better than that of the corresponding model, which did not include the random slope terms associated with the two kin-biased learning cues (*χ*^2^ = 45.5, *df* = 3, *p* < .001). As shown in **Table 3**, in addition to the mothers’ and fathers’ CREDs, the model in the third step revealed that the conformist learning cue was also associated with increased levels of religiosity (*b* = 0.34, 95% CI [0.17, 0.51], *t* = 3.83, *p* < .001). Thus, the average level of the religiosity score across countries or regions was 0.17 to 0.51 (average 0.34) points higher for a 1 *SD* increase in the percentage of older adults who showed religious CREDs. We also observed an interaction between the conformist learning cue and the mothers’ CREDs (*b* = −0.14, 95% CI [−0.24, −0.03], *t* = −2.50, *p* = .017). The association between the mothers’ CREDs and religiosity (*b* = 0.44, 95% CI [0.33, 0.56], *t* = 7.54, *p* < .001, VIF = 1.38) was weaker when the conformist learning cue was 1 *SD* higher (*b* = 0.31, 95% CI [0.17, 0.44], *t* = 4.55, *p* < .001) than when the cue was 1 *SD* lower (*b* = 0.58, 95% CI [0.40, 0.76], *t* = 6.37, *p* < .001). Although the interaction terms demonstrating the relationships between the conformist learning cue and the fathers’ CREDs did not reach statistical significance (*b* = −0.13, 95% CI [−0.26, 0.002], *t* = −1.94, *p* = .059), the 95% CI suggested that a 1 *SD* change in the conformist learning cue was associated with a decrease in the slope of the father’s CREDs. A simple slope analysis showed the same trend for the mothers’ CREDs. The coefficient of fathers’ CREDs (*b* = 0.57, 95% CI [0.43, 0.71], *t* = 7.99, *p* < .001, VIF = 1.38) became 0.44 when the conformist learning cue was 1 *SD* higher (95% CI [0.28, 0.60], *t* = 5.37, *p* < .001), whereas it was 0.69 when the cue was 1 *SD* lower (95% CI [0.48, 0.91], *t* = 6.32, *p* < .001). For each country or region, the betas of the parents’ CREDs are presented in **Fig 2**, with different color gradients indicating the conformist learning cue.

**Table 3:** The third-step model of hierarchical multilevel linear regression analysis for religiosity in the main focal group.

		95% confidence interval			
Predictors	Coefficient	Lower bound	Upper bound	*t*	*p*
Intercept	2.64	2.46	2.82	29.08	< .001
Gender	0.36	0.31	0.42	12.81	< .001
Mother’s CREDs	0.44	0.33	0.56	7.54	< .001
Father’s CREDs	0.57	0.43	0.71	7.99	< .001
Conformist learning cue	0.34	0.17	0.51	3.83	< .001
Mother’s CREDs * Conformist	–0.14	–0.24	–0.03	–2.50	.017
Father’s CREDs * Conformist	–0.13	–0.26	0.002	–1.94	.059
Random intercept variance	0.32				
Random slope variance					
Mother’s CREDs	0.03				
Father’s CREDs	0.06				

In short, the individual-level analyses conducted in this study demonstrated that kin-biased and conformist learning cues explained the individual differences in religious beliefs across different countries or regions. Interestingly, the results of these analyses also suggested that the associations between kin-biased learning cues and religious beliefs were robust when the conformist learning cue was not apparent. Regarding the main focal group, the results of the individual-level analyses demonstrated that the fathers’ religious behaviors had a greater impact on the religious beliefs of their children compared to the mothers’ religious behaviors.

### 3.2. International-level analyses

Next, we tested our hypotheses using an approach based on the signal detection theory. We calculated sensitivity (*d’*) and bias (*C*) using the parents’ religious CREDs as signals (0 = absent; 1 = present) and the measure for the belief in gods as the outcome (0 = absent; 1 = present). The predictions were that the average sensitivity index *d’* across countries would differ from zero, and the conformist learning cue would explain the bias index *C*. We examined these predictions separately using the mothers’ and the fathers’ CREDs.

#### 3.2.1. Mothers’ CREDs

**Fig 3:**
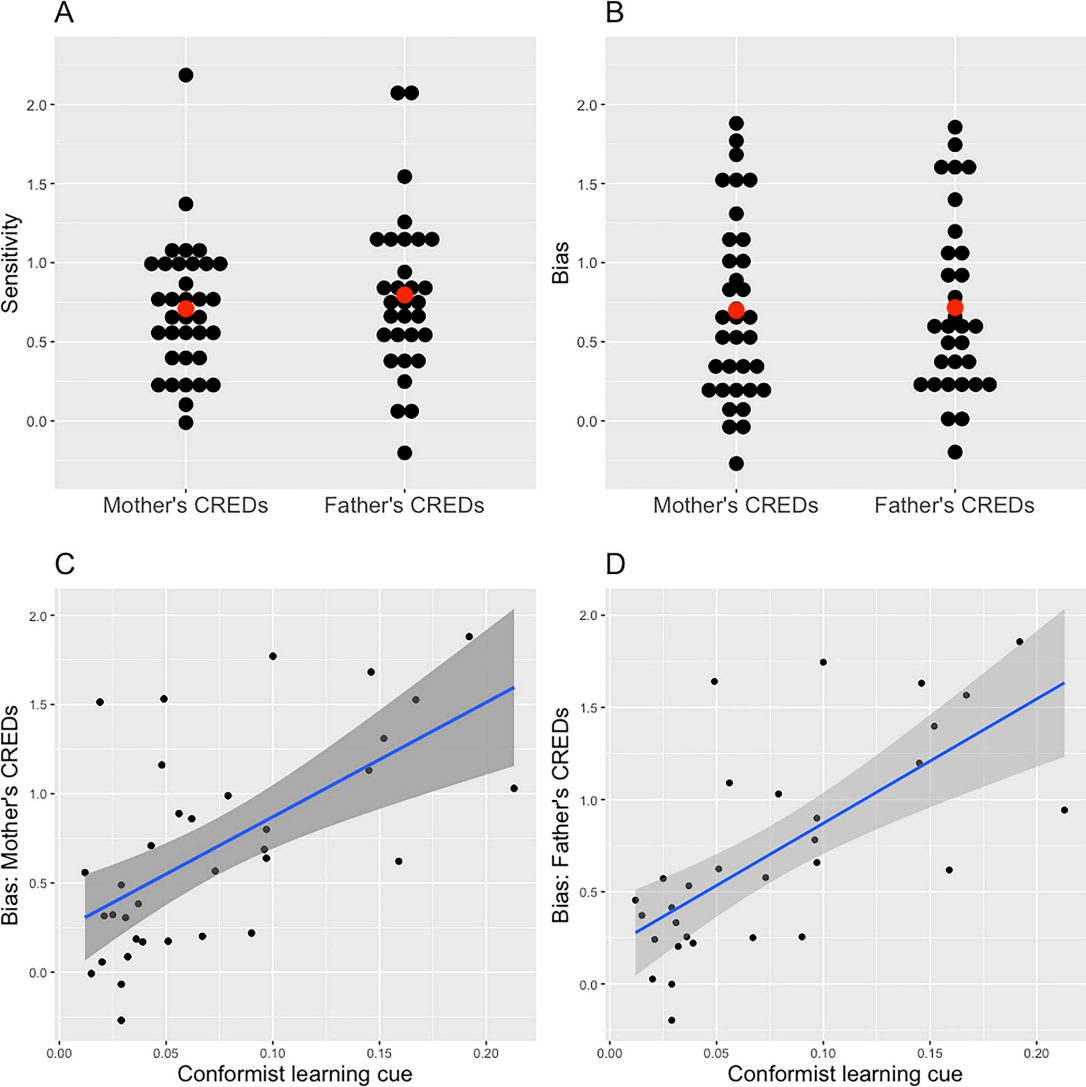
Response sensitivity (*d*’) and bias (*C*) toward mothers’ (A) and fathers’ CREDs (B) in the analysis of the data from the main focal group. The relationship between the conformist learning cue and response bias toward the mothers’ CREDs (C) and the fathers’ CREDs (D).

For each country or region, we calculated *d’* and *C* using the mothers’ religious CREDs. The countries or regions for which *d’* and/or *C* could not be calculated (e.g., where the mothers’ CREDs were not present among all the respondents in a country or region, the belief in gods was always present when the mothers’ CREDs were present) were excluded from the analysis. As predicted, a one-sample *t*-test (*t* (34) = 9.94, *p* < .001) and the 95% CI (0.56–0.85) showed that the average value of the index *d’* (*M* = 0.72, *SD* = 0.42) differed from zero (**Fig 3A**). This indicated that the respondents in the focal group were highly likely to believe in gods if they saw their mothers attending religious services at least once a week throughout their childhood. The average value of the index *C* (*M* = 0.70, *SD* = 0.58) was also likely to be above zero (95% CI [0.50, 0.90], *t* (34) = 7.15, *p* < .001, **Fig 3B**). We then conducted a regression analysis to test whether *C* could be explained by the conformist transmission cue after both variables were standardized. The t-test (*b* = 0.63, *t* = 4.47, *p* < .001) and the 95% CI (0.35–0.91) indicated that the conformist learning cue was positively related to bias toward the belief in gods (*R*_adj_^2^ = .358; **Fig 3C**), and this result suggested that people living in countries or regions with high percentages of religiously eager older adults tended to believe in gods.

#### 3.2.2. Fathers’ CREDs

We excluded 11 countries or regions for which *d’* and/or *C* could not be calculated, after which we conducted analyses for the fathers’ CREDs, which were similar to those conducted for the mothers’ CREDs. The average *d’* (*M* = 0.80, *SD* = 0.51, 95% CI [0.61, 0.98], *t* (30) = 8.65, *p* < .001) and *C* (*M* = 0.72, *SD* = 0.56, 95% CI [0.51, 0.92], *t* (30) = 7.08, *p* < .001) differed from zero (**Fig 3A and 3B**). Moreover, *C* was positively explained by the conformist learning cue (*b* = 0.68, 95% CI [0.42, 0.94], *t* = 5.08, *p* < .001, *R*_adj_^2^ = .453; **Fig 3D**).

In summary, the international-level analyses supported our predictions, thereby suggesting that the religious CREDs of parents and older adults independently affected the religious beliefs of the younger generation. The value of the index *d*’ associated with the fathers’ CREDs was slightly higher than that associated with the mothers’ CREDs, although the difference (−0.10) in the average *d*’ was not different from zero (95% CI [−0.21, 0.02], *t* (30) = 1.75, *p* = .090).

### 3.3. Additional analyses

Finally, as planned, we investigated the robustness of the present results by testing our hypotheses using data from respondents of different ages. We subsampled the respondents in the younger focal group (respondents born between 1981 and 1990, *N* = 7,632) and those in the older focal group (respondents born between 1961 and 1970, *N* = 9,433), after which we conducted analyses on their data, as described above. The figures and tables related to the additional analyses are presented in the Supporting Information.

#### 3.3.1. Younger focal group

The simple bivariable correlation matrix of the key variables in the younger focal group is shown in **S2 Table**.

We conducted hierarchical multilevel logistic regression analysis using the two kin-biased learning cues and the conformist learning cue to explain the belief in gods. The first-step model showed that the chances of believing in gods differed between different countries or regions (random intercept variance = 1.459, *χ*^2^ = 1744.6, *df* = 1, *p* < .001, ICC = .307). In the second step, we conducted analyses to determine whether the associations between the two kin-biased learning cues and the belief in gods varied between different countries or regions. Consequently, the second-step model assuming variations in these associations (i.e., the model with the two random slope terms) suggested a better fit than the corresponding model, which did not include variations. However, the differences in the deviance were not statistically significant (7709.2 vs. 7714.6, *χ*^2^ = 5.39, *df* = 2, *p* = .068). In the third step, the conformist learning cue and the interaction between it and the kin-biased learning cues on the belief in gods were entered (**S3 Table** and **S1 Fig**). This model revealed that the mothers’ CREDs (*b* = 1.08, OR = 2.94, 95% CI [2.24, 3.84], *z* = 7.87, *p* < .001, VIF = 1.31), the fathers’ CREDs (*b* = 0.61, OR = 1.83, 95% CI [1.39, 2.41], *z* = 4.34, *p* < .001, VIF = 1.34), and the conformist learning cue (*b* = 0.65, OR = 1.91, 95% CI [1.43, 2.55], *z* = 4.37, *p* < .001, VIF = 1.01) were independently associated with increased chances of believing in gods. However, the interaction effect was not observed (|*b*|s < 0.20, ORs < 1.20, |*z*|s < 1.54, *p*s > .120).

We also conducted hierarchical multilevel linear regression analysis using religiosity as the outcome variable. The correlation ratio (*η*^2^) between the belief in gods and religiosity was .576. The first-step model demonstrated between-country differences in religiosity (random intercept variance = 0.529, *χ*^2^ = 1662.8, *df* = 1, *p* < .001, ICC = .218), whereas the second-step model demonstrated between-country differences in the association between the presence of kin-biased learning cues and religiosity (*χ*^2^ = 107.75, *df* = 2, *p* < .001). The third-step model (**S4 Table** and **S2 Fig**) indicated that the mothers’ CREDs (*b* = 0.61, 95% CI [0.46, 0.76], *t* = 8.13, *p* < .001, VIF = 1.18), the fathers’ CREDs (*b* = 0.63, 95% CI [0.43, 0.84], *t* = 6.15, *p* < .001, VIF = 1.17), and the conformist learning cue (*b* = 0.28, 95% CI [0.09, 0.47], *t* = 2.86, *p* < .007, VIF = 1.01) were independently associated with religiosity. The conformist learning cue interacted with only the mothers’ CREDs (*b* = −0.28, 95% CI [−0.41, −0.15], *t* = −4.08, *p* < .001, VIF = 1.14) but not with the fathers’ CREDs (*b* = 0.02, 95% CI [−0.16, 0.21], *t* = 0.25, *p* = .803, VIF = 1.13). This result suggested that the mothers’ CREDs had a stronger association with the religiosity levels when the conformist learning cue was unclear (1 *SD* low: *b* = 0.89, 95% CI [0.67, 1.11], *t* = 7.92, *p* < .001) than when it was clear (1 *SD* high; *b* = 0.33, 95% CI [0.15, 0.50], *t* = 3.67, *p* = .002).

Next, we conducted analyses based on the signal detection theory (**S3 Fig**). We examined whether the average of the sensitivity index (*d*’) differed from zero and whether the bias index (*C*) was positively correlated with the conformist learning cue. One-sample *t*-tests and the 95% CI suggested that the average *d*’ associated with the mothers’ CREDs (*M* = 0.85, 95% CI [0.69, 1.01], *t* (37) = 10.61, *p* < .001) and the fathers’ CREDs (*M* = 0.74, 95% CI [0.57, 0.91], *t* (31) = 8.77, *p* < .001) were both higher than zero. The difference between the values of the two indices *d*’ was unlikely to be different from zero (95% CI [−0.04, 0.19], *t* (31) = 1.30, *p* = .204). Regression analyses showed that the conformist learning cue positively explained the index *C*, which was associated with the mothers’ CREDs (*b* = 0.60, 95% CI [0.30, 0.90], *t* = 3.96, *p* < .001, *R*_adj_^2^ = .284) and the fathers’ CREDs (*b* = 0.62, 95% CI [0.32, 0.93], *t* = 4.01, *p* < .001, *R*_adj_^2^ = .327).

#### 3.3.2. Older focal group

The simple bivariable correlation matrix of the key variables in the younger focal group is shown in **S5 Table**.

The correlation ratio (*η*^2^) between the belief in gods and religiosity in the older focal group was moderate (.580). The first steps of the hierarchical multilevel regression analyses revealed statistically significant between-country variations between the belief in gods (the random intercept variance = 1.35, *χ*^2^ = 1830, *df* = 1, *p* < .001, ICC = .291) and religiosity (random intercept variance = 0.454, *χ*^2^ = 1793.7, *df* = 1, *p* < .001, ICC = .199). The second-step model involving the belief in gods did not support the assumptions regarding country-by-country variations in the associations between the two kin-biased learning cues and the belief in gods (i.e., the deviance of the second-step model was not different from that of the corresponding model, which did not include between-country variations: *χ*^2^ = 1.59, *df* = 2, *p* = .451). However, the results of the third-step model were almost similar to those of the third-step models in the analyses of main and younger focal groups (**S6 Table** and **S4 Fig**). The mothers’ CREDs (*b* = 0.75, OR = 2.11, 95% CI [1.73, 2.57], *z* = 7.39, *p* < .001, VIF = 1.46), the fathers’ CREDs (*b* = 0.68, OR = 1.97, 95% CI [1.61, 2.41], *z* = 6.54, *p* < .001, VIF = 1.46), and the conformist learning cue (*b* = 0.68, OR = 1.97, 95%CI [1.52, 2.56], *z* = 5.06, *p* < .001, VIF = 1.01) were associated with the belief in gods. The interactions between the two types of learning cues were not likely (|*b*|s < 0.06, ORs < 1.06, |*z*|s < 0.60, *p*s > 0.59).

Meanwhile, the second-step model involving religiosity revealed between-country variations in the associations between the two kin-biased learning cues and religiosity (i.e., deviance of the second-step model significantly decreased from the corresponding model without between-country variations: *χ*^2^ = 31.0, *df* = 2, *p* < .001). In the third-step model, the mothers’ CREDs (*b* = 0.50, 95% CI [0.37, 0.62], *t* = 7.86, *p* < .001, VIF = 1.37), the fathers’ CREDs (*b* = 0.38, 95% CI [0.28, 0.49], *t* = 7.29, *p* < .001, VIF = 1.47), and the conformist learning cue (*b* = 0.34, 95% CI [0.18, 0.51], *t* = 4.12, *p* < .001, VIF = 1.01) were all associated with religiosity (**S7 Table** and **S5 Fig**). Additionally, the interaction between the mothers’ CREDs and the conformist learning cue was observed (*b* = −0.17, 95% CI [−0.29, −0.05], *t* = −2.86, *p* = .006, VIF = 1.30). This suggested that the association between the mothers’ CREDs and religiosity was greater when the conformist learning cue was unclear (−1 *SD*: *b* = 0.67, 95% CI [0.48, 0.86], *t* = 6.91, *p* < .001) than when it was clear (+ 1 *SD*; *b* = 0.32, 95% CI [0.17, 0.47], *t* = 4.16, *p* < .001). The interaction with the fathers’ CREDs was not observed (*b* = −0.03, 95% CI [−0.13, 0.06], *t* = −0.72, *p* = .481, VIF = 1.41).

The results of the analyses based on the signal detection theory were consistent with our predictions (**S6 Fig**). First, *d*’ associated with the mothers’ CREDs (*M* = 0.69, 95% CI [0.57, 0.81], *t* (35) = 11.68, *p* < .001) and the fathers’ CREDs (*M* = 0.73, 95% CI [0.61, 0.84], *t* (35) = 12.58, *p* < .001) both differed from zero. It is unlikely that these averages would be expected to differ (95% CI [−0.11, 0.02], *t* (34) = −1.26, *p* = .216). Second, the conformist learning cue explained the index of *C*, which was associated with the mothers’ CREDs (*b* = 0.58, 95% CI [0.28, 0.89], *t* = 3.72, *p* < .001, *R*_adj_^2^ = .269) and the fathers’ CREDs (*b* = 0.61, 95% CI [0.31, 0.91], *t* = 4.03, *p* < .001, *R*_adj_^2^ = .303).

### 3.4. Summary

The results of the primary and the additional analyses were mostly consistent. The kin-biased and conformist learning cues were associated with religious beliefs in the individual-level analyses. In these analyses, the aforementioned cues interacted. Specifically, the association between the mothers’ CREDs and religiosity increased when the conformist learning cue was ambiguous. The results of the international-level analyses also revealed that these cues were independently associated with the presence of the belief in gods.

However, there were differences between the results of the primary analyses and those of the additional analyses. First, the interaction between the two types of the cultural learning cues on the belief in gods was found only through the analysis of the data from the main focal group and not through the analysis of the data from the younger and older focal groups. Second, in the main focal group, religious beliefs were relatively more strongly associated with the fathers’ CREDs than with the mothers’ CREDs, whereas there were no differences in their associations, or possibly, their association with the mothers’ CREDs was stronger in the younger or older focal group. It is difficult to determine the reasons behind these differences because we do not have a theory or hypothesis explaining their occurrence. However, the results suggested that the associations between the two types of learning cues and religious beliefs vary depending on the samples and the generations involved. In this regard, conducting additional analyses was helpful. In other words, the additional analyses provided further insight into the associations observed across generations that are worth discussing. In the next section, we discuss the common results of the analyses conducted.

## 4. Discussion

The present study aimed to replicate and extend the findings of Gervais and Najle (2015)’s [32] study in which they demonstrated that religious beliefs are transmitted through kin-biased and conformist learning using data from the WVS, by focusing on CREDs in the cultural transmission of religious beliefs. Specifically, in this study, we analyzed data from the ISSP and examined whether kin-biased learning cues (i.e., the exposure to parental religious CREDs) and the conformist learning cue (i.e., the percentage of older generations who show religious CREDs in each country or region) were associated with the two measures of religious belief. The results of multilevel modeling as well as those of the analyses based on the signal detection theory clearly showed positive associations between the two types of learning cues and religious beliefs, and they were consistent with our predictions. Therefore, this study successfully replicated the findings of Gervais and Najle (2015)’s [32] study, and it provided additional evidence supporting the hypothesis that people acquire religious beliefs by modeling themselves after their parents and by conforming to societal trends.

The ISSP and WVS are well-known cross-cultural survey projects that have been used to test various hypotheses proposed in a wide range of studies associated with the field of social science. Although both survey projects involve similar items, they differ in terms of the countries or regions surveyed, the samples, as well as the content and format of the items involved. Consequently, the analysis of the data obtained from these large survey projects can yield different results, even when researchers use such data to test similar hypotheses [34]. Nevertheless, through this study, we obtained results that are consistent with those of a previous study, thereby demonstrating the robustness of the effects of kin-biased and conformist learning on the acquisition of religious beliefs.

These findings have implications for research in the cognitive science of religion. Since its inception, the cognitive science of religion has focused on the role of human cognitive functions in the acquisition of religious beliefs (e.g., [1]). Decades later, the crucial role of cultural transmission in the acquisition of religious beliefs has been garnering greater attention (e.g., [13, 35]). For example, previous studies have repeatedly suggested that caregivers’ CREDs was a good predictor of religious belief (e.g., [8, 12, 22–24]). Such studies mostly investigate the role of kin-biased transmission (e.g., the influence of parents’ CREDs). However, the role of conformist learning (e.g., the adoption of a religious belief based on its frequency) in the acquisition of religious beliefs has not been sufficiently investigated due to methodological constraints (i.e., difficulties in estimating the number of people engaging in religious behavior in a standard survey method). Based on this context, Gervais and Najle (2015) [32] overcame these methodological constraints by exploring the association between the acquisition of religious beliefs among individuals in the younger generation and the number of older adults who engage in enthusiastic religious activities in various countries or regions. They evaluated the validity of their hypotheses by analyzing data obtained from the WVS, and they obtained satisfactory results. In this study, we confirmed the robustness of their results. The results of this study will encourage scholars specializing in the cognitive science of religion to recognize the importance of cultural transmission in the acquisition of religious beliefs, thereby encouraging further research in this field.

### 4.1. Differences in results from Gervais and Najle (2015)

In addition to the datasets analyzed in this study, there were some other differences between the results of this study and those obtained by Gervais and Najle (2015) [32]. Discussing these differences will help clarify the implications of this study.

First, we regarded the parents’ religious CREDs as the kin-biased learning cues, instead of following the approach employed by Gervais and Najle (2015) [32], who used religious upbringing. We used this approach for practical reasons (i.e., items about religious upbringing are not included in the ISSP questionnaire) as well as theoretical reasons. According to the theory of CREDs [14], it can be hypothesized that parents’ CREDs have a greater impact on their children’s religious beliefs, and previous empirical studies have demonstrated that religious CREDs are better predictors of religious beliefs than religious upbringing [22]. Consistent with these findings, this study revealed an association between parents’ CREDs and the acquisition of religious beliefs among their children, thereby suggesting that parents’ CREDs influenced their children’s religious beliefs. Therefore, this study successfully demonstrated the importance of CREDs in kin-biased learning.

Second, we created two kin-biased learning cues (the mothers’ and the fathers’ CREDs) and compared them in terms of their associations with religious beliefs. Focusing on the simple association between each cue and religious beliefs, the results showed no consistent trends across generations (i.e., analyses of the main, younger, and older focal groups). Therefore, we could not draw a clear conclusion from the results. It should be noted that the results were not consistent with those obtained by Cavalli-Sforza et al. (1982) [25], which are sometimes cited as evidence of the dominance of maternal influence on the acquisition of religious beliefs [36]. This lack of consistency probably originates from the samples and the measures for religious belief used in these studies. Cavalli-Sforza et al. (1982) [25] conducted a study in the United States in which they asked university students (*N* = 203) and their parents whether they were affiliated with Christianity or Judaism, as well as the frequency at which they attended church and prayed. They then assessed the extent to which the students’ responses were associated with those of their mothers and fathers. The results revealed that the affiliation to and the frequency of prayer among the students were more strongly associated with their mothers’ responses than with their fathers’ responses. The students’ church attendance frequencies were equally associated with both responses. However, in this study, we used larger and highly diverse samples (i.e., thousands of people from various countries or regions), and the measures for religious belief are more direct (i.e., whether the respondents believe in gods and whether they consider themselves religious). Therefore, the results of this study were highly representative of global trends. It should also be noted that the results of this study are not conclusive, and Cavalli-Sforza et al. (1982) [25] should be appreciated because their findings demonstrated the significance of vertical transmission in the acquisition of religious beliefs. Further research is required to determine whether mothers or fathers influence the religious beliefs of their children by considering the differences in age, region, and religious culture.

The third difference involved the outcome variable. Gervais and Najle (2015) [32] used only the belief in gods as the outcome variable. Contrarily, we used an item for measuring religiosity (whether the respondents considered themselves religious) in addition to an item for measuring the belief in gods (whether the respondents believed in gods). These two items have sometimes been bundled together and used to measure similar religious beliefs (e.g., [12]), but they have different aspects. The item for assessing the belief in gods item assesses whether people are sure about the existence of supernatural agents, whereas the item for measuring religiosity reflects not only such beliefs but also whether people value religious ideas and practices in their lives. Therefore, the item for assessing religiosity is a measure of religious beliefs in a broader sense. The results of our analyses revealed that the scores on these items were similarly associated with the two cultural learning cues. Hence, this study successfully demonstrated the generalizability of Gervais and Najle (2015)’s [32] findings in terms of assessing religious beliefs.

Moreover, we found differences in the associations between the two types of cultural learning cues and the two measures. The kin-biased and conformist learning cues interacted in the analyses, with religiosity being the outcome variable. This interaction suggested tension between the effects of the two cultural learning cues on religiosity; mothers’ religious CREDs more strongly influence their children’s religiosity in countries or regions where fewer older adults exhibit religious CREDs, and they have a weaker influence in places where a high percentage of older adults exhibit religious CREDs. This result is of interest for several reasons. First, it suggests a boundary condition for the effects of kin-biased learning on the acquisition of religious beliefs. Previous studies that investigated the effects of cultural learning on the acquisition of religious beliefs mainly focused on vertical transmission (e.g., parents’ CREDs). However, the effects of vertical transmission could be moderated by other transmission pathways, such as horizontal and oblique transmissions, including conformist learning. It would be helpful to consider the extent to which horizontal and oblique transmissions are expected when evaluating the findings of previous studies examining the effects of parents’ religious behaviors on their children’s religious beliefs. Second, the results indicated the possibility that the effects of cultural transmission differ depending on the aspects of the religious beliefs involved. The interaction between the two cultural learning cues was consistently observed primarily with regard to the measure of religiosity (i.e., beliefs in the broader sense), but not regarding the belief in gods. Therefore, the influence of cultural transmission (e.g., kin-biased learning) on religiosity could be modulated by other learning pathways (e.g., conformist learning), whereas its effects on the belief in gods might remain unmodulated. Examining this idea as well as its theoretical implications in future research may be worthwhile. Finally, only the mothers’ CREDs consistently interacted with the conformist learning cue across generations. Considering the findings of previous studies on the relationship between parental influences and the acquisition of religious beliefs, this is an interesting result. According to the findings demonstrating that maternal transmission is more influential in the acquisition of religious beliefs [25], the father’s influence is expected to be attenuated by other transmission pathways. However, the results of this study argue otherwise. They suggest that which parents are more influential in their children’s religious beliefs depends on the society in which people live or their culture. Therefore, further research on the ways in which parents’ behaviors influence their children’s religious beliefs would be helpful.

### 4.2. Limitations

Lastly, some limitations of the present study should be acknowledged. First, this study focused on CREDs in cultural transmission to explain the individual differences in religious beliefs. However, there are other factors, such as cognitive abilities and secularization that have been shown to explain individual differences (e.g., [28]). We did not compare CREDs with these factors in terms of their association with religious beliefs. Thus, this study only demonstrated the association between parental and elderly CREDs and religious beliefs. Future studies comparing various factors will help us better understand how individual differences in religious beliefs emerge. Related to this point, this study only examined the role of CREDs in cultural transmission in the religious domain, although it is hypothesized that CREDs function in other domains (e.g., food preferences, altruism). Investigating whether similar results are observed in other cultural domains would be an interesting way to understand the role of CREDs in cultural transmission.

Second, this study examined the relationship between CREDs and beliefs only in the domain of religion. However, the theory of CREDs [14] has been applied not only to the domain of religion, but also to a variety of cultural representations (e.g., food preferences, opinions about the social system, and altruism). This raises the question of whether similar results to those of this study can be observed in another cultural domain. For example, the ISSP covered various topics, such as work, health, and sports. Comparing the effect of CREDs on beliefs, norms, or preferences in different domains is an interesting area for future research.

Third, although this study succeeded in suggesting the effects of kin-biased and conformist learning cues on the acquisition of religious beliefs, the methods employed were purely correlational. Further experimental studies are required to demonstrate such effects. However, it may be challenging to strengthen or weaken religious beliefs in experimental contexts because such an approach could raise ethical concerns. Therefore, it is useful to deepen our understanding of the cultural transmission of religious beliefs by referring to findings involving the horizontal, vertical, and oblique transmission of other types of cultural information.

Finally, the two cultural learning cues used in this study adequately demonstrated the possibility of the cultural transmission of religious beliefs. However, they remained admittedly imperfect. Therefore, the results of this study are just preliminary. It is necessary to examine the cultural learning of religious beliefs by creating other indices. For example, we created our cultural leaning cues by dichotomizing the responses so that the cues were consistent with the definition of CREDs. It is also due to methodological convenience (e.g., signal detection approach). However, CREDs are a "more or less” phenomenon, rather than a "present or absent" one. Thus, our cultural learning cues may have been oversimplified indices of CREDs, and the use of another index would be helpful. Moreover, the kin-biased learning cues used in this study were created based on the respondents’ retrospective answers to questions about their parents’ behaviors when they were children. It would be useful to collect data directly from the respondents’ parents. Additionally, the conformist learning cue used in this study captured only one aspect of religious CREDs: the frequency of participation in religious activities or organizations. It is worth examining conformist learning by considering other behaviors (e.g., the frequency of praying, religious practices, or wearing religious items). In addition, according to the cultural evolutionary theory [36], conformist learning in the acquisition of religious beliefs suggests that people tend to be disproportionately highly likely to adopt the religious beliefs of the older group than to copy them randomly. In this study, we followed the methods employed by Gervais and Najle (2015) [32], and we found a robust association between the cue for conformity learning and religious beliefs. However, it still remains unclear whether the association captured the tendencies involved. To determine the effects of conformity learning on the acquisition of religious beliefs, it is helpful to investigate whether religious beliefs (or disbeliefs) spread as predicted by the cultural evolutionary theory. While overcoming these limitations, further studies should test the robustness of the results, particularly those of the exploratory analysis; the interaction between kin-biased learning and conformist learning. In addition, it is essential to develop a theory of multiple competing cultural learning pathways.

### Notes

1. The item “Were you brought up religiously at home?” is only in WVS 2 and 3. Thus, Gervais and Najle (2015) [32] analyzed datasets obtained from WVS 2 and/or 3.
2. All the participants from Venezuela were excluded from the study owing to missing responses to the two items used to create the measures for the kin-biased learning cues.
3. According to Stanislaw and Todorov (1999) [37], the value of *d*’ was calculated by subtracting the *z* score corresponding to the false-alarm rate from the *z* score corresponding to the hit rate. The value of *C* was calculated by averaging the *z* score corresponding to the hit rate and the z score corresponding to the false-alarm rate. The values of *d*’ and *C* will be zero if there is no sensitivity to a signal and response bias.
4. The coefficient of the logistic regression analysis indicates how much the log-odds of the outcome variable (e.g., presence of belief in gods) change with a one-point increase in the predictor variable (e.g., the cultural learning cue; the percentage of the respondents in the older group who showed religious CREDs). The range of our cultural learning cue among 42 countries or regions was 0.20 (*M* = 0.07, *SD* = 0.06), and no country or region had a value of 1 on this cue. Thus, the 1-point increase in this cue was impractical, and in this case, the coefficients would be difficult to interpret. This is why we employed standardization for the conformist learning cue; the coefficient can be interpreted as how much the log-odds of the presence of the belief in gods change with a 1 *SD* increase in the predictor variable.
5. The phi coefficient between the mothers’ and the fathers’ CREDs was .706 when ignoring a country or region. When the phi coefficients were calculated for each country or region, the mean was .685 (*SD* = 0.148). In some countries, the two cues matched perfectly. For instance, all the parents of the respondents from East Germany showed religious CREDs, whereas the parents of the respondents from Japan and Russia did not show religious CREDs at all. These results indicated high levels of similarity in parents’ behaviors, thereby raising concerns about the problem of multicollinearity. However, the VIFs of the two CREDs in the analytical models were acceptable. Therefore, both variables were entered simultaneously, thereby prioritizing the advantages of comparing the effects of the two CREDs.

## Supporting information

S1 Fig The association between kin-biased learning cues and the belief in gods (odds ratios) across 42 countries or regions in the analysis of the data from the younger focal group. (PDF)

S2 Fig The association between kin-biased learning cues and religiosity (beta) across 42 countries or regions in the analysis of the data from the younger focal group. (PDF)

S3 Fig Response sensitivity (*d*’) and bias (*C*) toward mothers’ (A) and fathers’ CREDs (B) in the analysis of the data from the younger focal group. The relationship between the conformist learning cue and response bias toward the mothers’ CREDs (C) and the fathers’ CREDs (D).
(PDF)

S4 Fig The association between kin-biased learning cues and the belief in gods (odds ratios) across 42 countries or regions in the analysis of the data from the older focal group. (PDF)

S5 Fig The association between kin-biased learning cues and religiosity (beta) across 42 countries or regions in the analysis of the data from the older focal group. (PDF)

S6 Fig Response sensitivity (*d*’) and bias (*C*) toward mothers’ (A) and fathers’ CREDs (B) in the analysis of the data from the older focal group. The relationship between the conformist learning cue and response bias toward the mothers’ CREDs (C) and the fathers’ CREDs (D).
(PDF)

S1 Table The bivariable correlation matrix of the key variables in the main focal group. (PDF)

S2 Table The bivariable correlation matrix of the key variables in the younger focal group. (PDF)

S3 Table The third-step model of hierarchical multilevel logistic regression analysis for the belief in gods in the younger focal group. (PDF)

S4 Table The third-step model of hierarchical multilevel linear regression analysis for religiosity in the younger focal group. (PDF)

S5 Table The bivariable correlation matrix of the key variables in the older focal group. (PDF)

S6 Table The third-step model of hierarchical multilevel logistic regression analysis for the belief in gods in the older focal group. (PDF)

S7 Table The third-step model of hierarchical multilevel linear regression analysis for religiosity in the older focal group. (PDF)
