# Supplementary material for: Cultural transmission and religious belief: An extended replication of Gervais and Najle (2015) using data from the International Social Survey Programme
Source: PLoS One. 2024 Jun 24;19(6):e0305635. doi: 10.1371/journal.pone.0305635 (PMC11195988; doi:10.1371/journal.pone.0305635)

S4 Fig. The association between the kin-biased learning cues and the belief in gods (odds ratios) across 42 countries or regions in the analysis of the data from the older focal group. The dotted line indicates the fixed slope of the kin-biased learning cue in the overall sample. Color gradients indicate the conformist learning (CL) cue in each country or region. The horizontal axis indicates the odds ratio of kin-biased learning cues on a log scale.

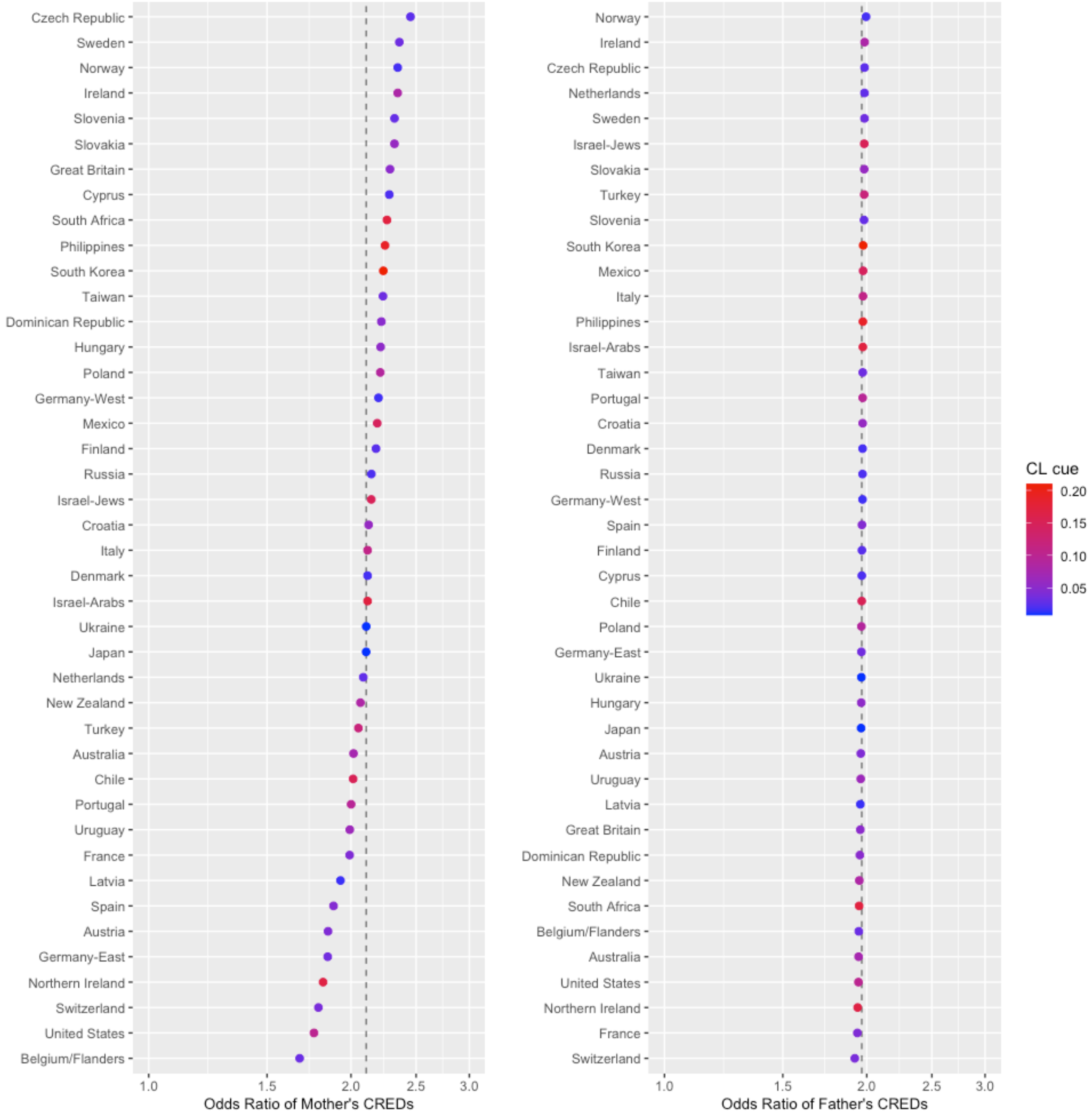

Supplement: S4 Fig — (PDF) [file pone.0305635.s004.pdf]
