# Supplementary material for: Cultural transmission and religious belief: An extended replication of Gervais and Najle (2015) using data from the International Social Survey Programme
Source: PLoS One. 2024 Jun 24;19(6):e0305635. doi: 10.1371/journal.pone.0305635 (PMC11195988; doi:10.1371/journal.pone.0305635)

**S6 Fig. Response sensitivity (*d*') and bias (*C*) toward mothers' (A) and fathers' CREDs (B) in the analysis of the data from the older focal group. The relationship between the conformist learning cue and response bias toward the mothers' CREDs (C) and the fathers' CREDs (D).**

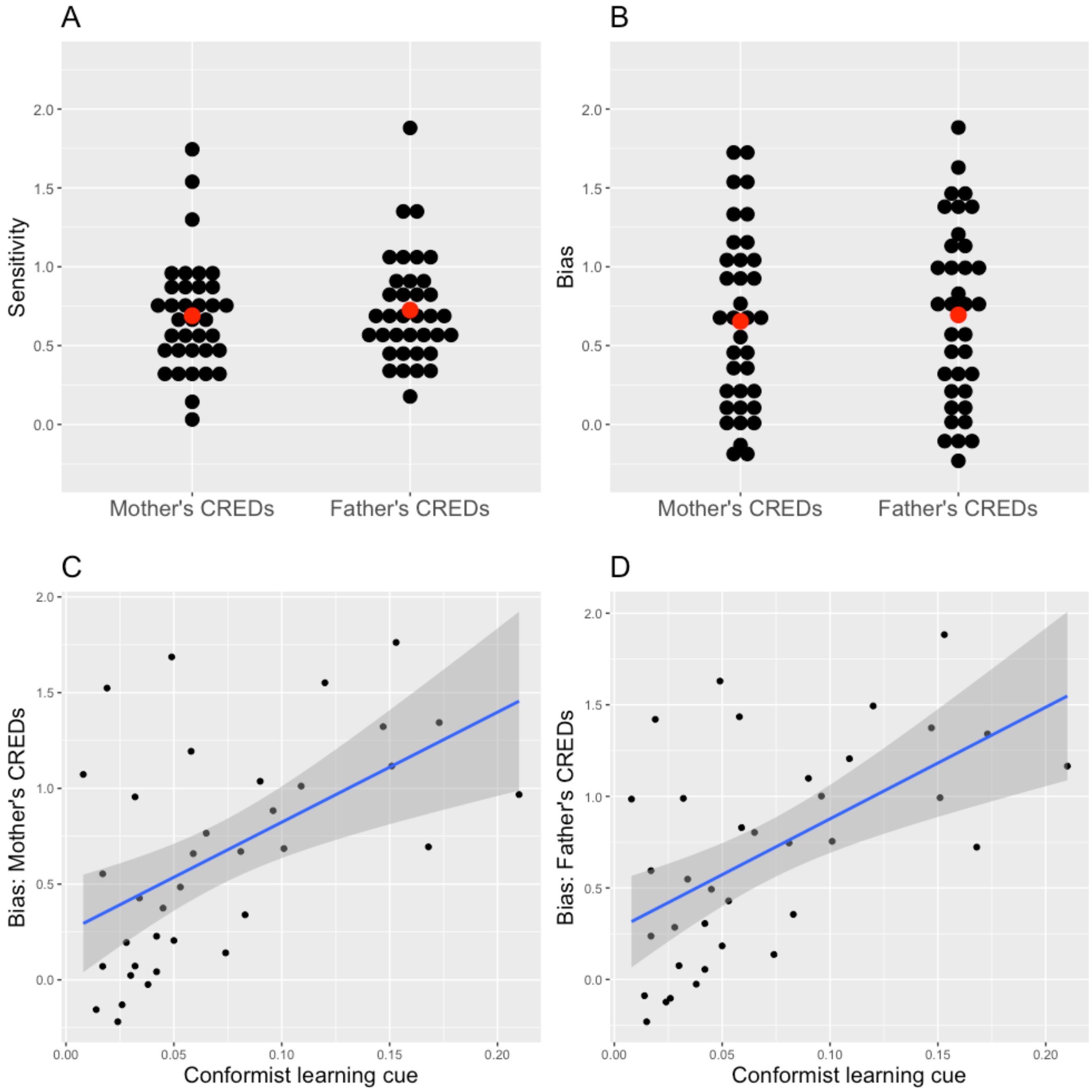

Supplement: S6 Fig — Response sensitivity (d’) and bias (C) toward mothers’ (A) and fathers’ CREDs (B) in the analysis of the data from the older focal group. The relationship between the conformist learning cue and response bias toward the mothers’ CREDs (C) and the fathers’ CREDs (D). (PDF) [file pone.0305635.s006.pdf]
