# Supplementary material for: Cultural transmission and religious belief: An extended replication of Gervais and Najle (2015) using data from the International Social Survey Programme
Source: PLoS One. 2024 Jun 24;19(6):e0305635. doi: 10.1371/journal.pone.0305635 (PMC11195988; doi:10.1371/journal.pone.0305635)
Supplement: S1 Table — (PDF) [file pone.0305635.s007.pdf]

S1 Table. The bivariable correlation matrix of the key variables in the main focal group.

	Belief in gods (6-points)	Belief in gods (dichotomised)	Religiosity (7-points)	Mother's attendance (dichotomised)	Father's attendance (dichotomised)	Mother's attendance (9-points)	Father's attendance (9-points)	Conformist learning cue	Gender (0 = M, 1 = F)
Belief in gods (6-points)	-	.897	.658	.286	.283	.420	.399	.330	.114
Belief in gods (dichotomised)		-	.577	.246	.242	.368	.344	.280	.091
Religiosity (7-points)			-	.277	.280	.433	.429	.257	.119
Mother's attendance (dichotomised)				-	.706	.758	.588	.255	.011
Father's attendance (dichotomised)					-	.559	.762	.238	.010
Mother's attendance (9-points)						-	.752	.310	.018
Father's attendance (9-points)							-	.286	-.001
Conformist learning cue								-	.031
Gender (0 = M, 1 = F)									-

* Note that the nested structure of the data in this study is not reflected in this correlation matrix.
