# Supplementary material for: Cultural transmission and religious belief: An extended replication of Gervais and Najle (2015) using data from the International Social Survey Programme
Source: PLoS One. 2024 Jun 24;19(6):e0305635. doi: 10.1371/journal.pone.0305635 (PMC11195988; doi:10.1371/journal.pone.0305635)
Supplement: S2 Table — (PDF) [file pone.0305635.s008.pdf]

S2 Table. The bivariable correlation matrix of the key variables in the younger focal group.

	Belief in gods (6-points)	Belief in gods (dichotomised)	Religiosity (7-points)	Mother's attendance (dichotomised)	Father's attendance (dichotomised)	Mother's attendance (9-points)	Father's attendance (9-points)	Conformist learning cue	Gender (0 = M, 1 = F)
Belief in gods (6-points)	-	.901	.654	.309	.292	.452	.431	.314	.107
Belief in gods (dichotomised)		-	.576	.273	.249	.408	.377	.278	.081
Religiosity (7-points)			-	.293	.295	.462	.452	.228	.100
Mother's attendance (dichotomised)				-	.682	.752	.565	.253	-.002
Father's attendance (dichotomised)					-	.540	.753	.243	-.005
Mother's attendance (9-points)						-	.731	.311	.001
Father's attendance (9-points)							-	.288	-.007
Conformist learning cue								-	-.015
Gender (0 = M, 1 = F)									-

* Note that the nested structure of the data in this study is not reflected in this correlation matrix.
