# Supplementary material for: Cultural transmission and religious belief: An extended replication of Gervais and Najle (2015) using data from the International Social Survey Programme
Source: PLoS One. 2024 Jun 24;19(6):e0305635. doi: 10.1371/journal.pone.0305635 (PMC11195988; doi:10.1371/journal.pone.0305635)
Supplement: S3 Table — (PDF) [file pone.0305635.s009.pdf]

S3 Table. The third-step model of hierarchical multilevel logistic regression analysis for the belief in gods in the younger focal group.

		95% confidence interval			
Predictors	Odds ratio	Lower bound	Upper bound	*z*	*p*
Intercept	1.10	0.82	1.47	0.61	.540
Gender	1.53	1.37	1.71	7.43	< .001
Mother's CREDs	2.94	2.24	3.84	7.87	< .001
Father's CREDs	1.83	1.39	2.41	4.34	< .001
Comformist learning cue	1.91	1.43	2.55	4.37	< .001
Mother's CREDs * Conformist	0.82	0.64	1.06	-1.54	.124
Father's CREDs * Conformist	1.19	0.90	1.57	1.23	.220
Random intercept variance	0.84				
Random slope variance					
Mother's CREDs	0.15				
Father's CREDs	0.07				
