# Supplementary material for: Cultural transmission and religious belief: An extended replication of Gervais and Najle (2015) using data from the International Social Survey Programme
Source: PLoS One. 2024 Jun 24;19(6):e0305635. doi: 10.1371/journal.pone.0305635 (PMC11195988; doi:10.1371/journal.pone.0305635)
Supplement: S4 Table — (PDF) [file pone.0305635.s010.pdf]

S4 Table. The third-step model of hierarchical multilevel linear regression analysis for religiosity in the younger focal group.

		95% confidence interval			
Predictors	Coefficient	Lower bound	Upper bound	*t*	*p*
Intercept	2.47	2.27	2.67	24.15	< .001
Gender	0.31	0.25	0.37	10.09	< .001
Mother's CREDs	0.61	0.46	0.76	8.13	< .001
Father's CREDs	0.63	0.43	0.84	6.15	< .001
Comformist learning cue	0.28	0.09	0.47	2.86	< .001
Mother's CREDs * Conformist	-0.28	-0.41	-0.15	-4.08	< .001
Father's CREDs * Conformist	0.02	-0.16	0.21	0.25	.803
Random intercept variance	0.41				
Random slope variance					
Mother's CREDs	0.08				
Father's CREDs	0.21				
