# Supplementary material for: Cultural transmission and religious belief: An extended replication of Gervais and Najle (2015) using data from the International Social Survey Programme
Source: PLoS One. 2024 Jun 24;19(6):e0305635. doi: 10.1371/journal.pone.0305635 (PMC11195988; doi:10.1371/journal.pone.0305635)
Supplement: S5 Table — (PDF) [file pone.0305635.s011.pdf]

S5 Table. The bivariable correlation matrix of the key variables in the older focal group.

	Belief in gods (6-points)	Belief in gods (dichotomised)	Religiosity (7-points)	Mother's attendance (dichotomised)	Father's attendance (dichotomised)	Mother's attendance (9-points)	Father's attendance (9-points)	Conformist learning cue	Gender (0 = M, 1 = F)
Belief in gods (6-points)	-	.900	.672	.296	.277	.397	.380	.327	.110
Belief in gods (dichotomised)		-	.580	.254	.238	.351	.333	.286	.088
Religiosity (7-points)			-	.268	.262	.393	.379	.258	.118
Mother's attendance (dichotomised)				-	.711	.774	.609	.231	.011
Father's attendance (dichotomised)					-	.569	.772	.224	.004
Mother's attendance (9-points)						-	.754	.271	-.005
Father's attendance (9-points)							-	.258	-.020
Conformist learning cue								-	.021
Gender (0 = M, 1 = F)									-

* Note that the nested structure of the data in this study is not reflected in this correlation matrix.
