# Supplementary material for: Cultural transmission and religious belief: An extended replication of Gervais and Najle (2015) using data from the International Social Survey Programme
Source: PLoS One. 2024 Jun 24;19(6):e0305635. doi: 10.1371/journal.pone.0305635 (PMC11195988; doi:10.1371/journal.pone.0305635)
Supplement: S6 Table — (PDF) [file pone.0305635.s012.pdf]

S6 Table. The third-step model of hierarchical multilevel logistic regression analysis for the belief in gods in the older focal group.

		95% confidence interval			
Predictors	Odds ratio	Lower bound	Upper bound	*z*	*p*
Intercept	1.32	1.01	1.73	2.06	.040
Gender	1.61	1.46	1.78	9.44	< .001
Mother's CREDs	2.11	1.73	2.57	7.39	< .001
Father's CREDs	1.97	1.61	2.41	6.54	< .001
Comformist learning cue	1.97	1.52	2.56	5.06	< .001
Mother's CREDs * Conformist	1.06	0.86	1.31	0.53	.597
Father's CREDs * Conformist	0.97	0.78	1.21	-0.26	.793
Random intercept variance	0.69				
Random slope variance					
Mother's CREDs	0.04				
Father's CREDs	0.004				
