# Supplementary material for: Cultural transmission and religious belief: An extended replication of Gervais and Najle (2015) using data from the International Social Survey Programme
Source: PLoS One. 2024 Jun 24;19(6):e0305635. doi: 10.1371/journal.pone.0305635 (PMC11195988; doi:10.1371/journal.pone.0305635)
Supplement: S7 Table — (PDF) [file pone.0305635.s013.pdf]

S7 Table. The third-step model of hierarchical multilevel linear regression analysis for religiosity in the older focal group.

		95% confidence interval			
Predictors	Coefficient	Lower bound	Upper bound	*t*	*p*
Intercept	2.75	2.58	2.92	32.20	< .001
Gender	0.35	0.30	0.41	12.88	< .001
Mother's CREDs	0.50	0.37	0.62	7.86	< .001
Father's CREDs	0.38	0.28	0.49	7.29	< .001
Comformist learning cue	0.34	0.18	0.51	4.12	< .001
Mother's CREDs * Conformist	-0.17	-0.29	-0.05	-2.86	.006
Father's CREDs * Conformist	-0.03	-0.13	0.06	-0.72	.481
Random intercept variance	0.28				
Random slope variance					
Mother's CREDs	0.06				
Father's CREDs	0.004				
